# First-in-human diagnostic study of hepatic steatosis with computed ultrasound tomography in echo mode

**DOI:** 10.1038/s43856-023-00409-3

**Published:** 2023-12-09

**Authors:** Patrick Stähli, Chiara Becchetti, Naiara Korta Martiartu, Annalisa Berzigotti, Martin Frenz, Michael Jaeger

**Affiliations:** 1 Institute of Applied Physics, University of Bern, Bern, Switzerland; 2 Department of Visceral Surgery and Medicine, Inselspital, Bern University Hospital, University of Bern, Bern, Switzerland; 3 Department of Biomedical Research, University of Bern, Bern, Switzerland

**Keywords:** Diagnostic markers, Metabolic disorders, Diagnostic markers, Ultrasonography, Diagnosis

## Abstract

**Background:**

Non-alcoholic fatty liver disease is rapidly emerging as the leading global cause of chronic liver disease. Efficient disease management requires low-cost, non-invasive techniques for diagnosing hepatic steatosis accurately. Here, we propose quantifying liver speed of sound (SoS) with computed ultrasound tomography in echo mode (CUTE), a recently developed ultrasound imaging modality adapted to clinical pulse-echo systems. CUTE reconstructs the spatial distribution of SoS by measuring local echo phase shifts when probing tissue at varying steering angles in transmission and reception.

**Methods:**

In this first-in-human phase II diagnostic study, we evaluated the liver of 22 healthy volunteers and 22 steatotic patients. We used conventional B-mode ultrasound images and controlled attenuation parameter (CAP) to diagnose the presence (CAP≥ 280 dB/m) or absence (CAP < 248 dB/m) of steatosis in the liver. A fully integrated convex-probe CUTE implementation was developed on the ultrasound system to estimate liver SoS. We investigated its diagnostic value via the receiver operating characteristic (ROC) analysis and correlation to CAP measurements.

**Results:**

We show that liver CUTE-SoS estimates correlate strongly (*r* = −0.84, *p* = 8.27 × 10^−13^) with CAP values and have 90.9% (95% confidence interval: 84–100%) sensitivity and 95.5% (81–100%) specificity for differentiating between normal and steatotic livers (area under the ROC curve: 0.93–1.0).

**Conclusions:**

Our results demonstrate that liver CUTE-SoS is a promising quantitative biomarker for diagnosing liver steatosis. This is a necessary first step towards establishing CUTE as a new quantitative add-on to diagnostic ultrasound that can potentially be as versatile as conventional ultrasound imaging.

## Plain language summary

Non-alcoholic fatty liver disease (NAFLD), characterized by fat accumulation in the liver, is rapidly becoming the most common cause of chronic liver disease worldwide. Therefore, there is an urgent need to develop accurate diagnostic techniques that are inexpensive, non-invasive, and broadly available. Ultrasound imaging systems, which use sound waves to produce images of internal body structures, possess these qualities but cannot currently diagnose NAFLD accurately. Here, we propose to use a recently developed technique called computed ultrasound tomography in echo mode (CUTE). It measures the speed at which ultrasound waves propagate in tissues, a property that substantially varies with the fat content. We show that CUTE measurements allow us to accurately distinguish the livers of healthy people from those of individuals diagnosed with NAFLD. This promising finding encourages the integration of CUTE into standard ultrasound systems.

## Abstract (web summary)

Stähli, Becchetti, Korta Martiartu et al. present a first-in-human evaluation of computed ultrasound tomography in echo mode to quantify the speed of sound in the liver. Estimated values show an excellent discriminative ability in distinguishing normal versus steatotic livers, with potential value in the non-invasive diagnosis of liver steatosis.

## Introduction

Non-alcoholic fatty liver disease (NAFLD) is currently the most prevalent type of chronic liver disease, with an estimated number of one billion affected individuals worldwide^1–3^. NAFLD is characterized by steatosis, namely the accumulation of fat in the liver, and its aggressive form (non-alcoholic steatohepatitis) may progress to liver fibrosis, cirrhosis, and eventually hepatocellular carcinoma^4^. Early diagnosis of hepatic steatosis is crucial to identify subjects at risk of developing advanced liver diseases, as well as cardiovascular and metabolic diseases^5,6^. In this regard, liver biopsy and magnetic resonance (MR) are the gold standards, although both become inefficient and inadequate for large-scale screening that the increasing prevalence of NAFLD requires^7^. Liver biopsy is an expensive and invasive technique limited by sampling error and associated risk of complications^8,9^. MR is non-invasive, but the high cost and limited availability of MR systems restrict the use of this technique in clinical practice^7^. Developing cost-effective, non-invasive diagnostic methods is therefore a high clinical and research priority.

Ultrasound (US) imaging systems possess essential qualities to tackle the challenges of screening for NAFLD. They are safe, portable, relatively inexpensive, readily available in most clinical facilities, and provide real-time feedback. Thus, US is a more convenient imaging modality than MR for large-scale screening. In fact, US is commonly employed to assess hepatic steatosis by analyzing features in conventional gray-scale B-mode images such as the echo intensity, distribution, and contrast^10,11^. Except for the hepatorenal index, these parameters are qualitative, and their assessment strongly depends on the expertise of the sonographer^12^. Such subjective evaluation makes the sensitivity of conventional US particularly poor (60.9–65%) for low-grade hepatic steatosis (≤20% fat content)^10,13,14^.

Quantitative indicators of steatosis are indispensable to improving the sensitivity and diagnostic accuracy of US. The fat accumulation alters the liver composition and, consequently, its acoustic and mechanical properties, which we can quantify from US radio-frequency signals. For example, the energy loss or attenuation that US waves undergo during their propagation through the liver increases in the presence of fat^15^ and can be used as an indicator of steatosis. With this idea in mind, the controlled attenuation parameter (CAP) has been developed using vibration-controlled transient elastography on FibroScan equipment (Echosens, Paris, France)^16^. CAP estimates the average US attenuation in a cylindrical volume of the liver of approximately 1 cm diameter at 25-65 mm depth using 3.5 MHz M probes (or at 35–75 mm depth with 2.5 MHz XL probes)^17^ and shows significant correlations with histological steatosis grades^16,18–20^. Despite emerging as a widely used non-invasive diagnostic tool, CAP measurements depend substantially on covariates such as etiology, diabetes status, or body mass index, bringing concerns about its diagnostic performance^21–23^.

Quantifying other tissue properties sensitive to composition could complement CAP measurements to improve its diagnostic ability. A promising candidate is the propagation velocity of longitudinal US waves in tissue, referred to as the speed of sound (SoS), which decreases considerably with fat content^24–26^. A recent clinical study in a population with different grades of steatosis has shown encouraging results with excellent correlations between average liver SoS estimates and MR-based fat-fraction measurements^27^. However, these average SoS measurements do not capture the intrinsic tissue heterogeneity and require a manual correction with assumed values of the SoS distribution in the abdominal wall^25,28^, making them prone to biases. This limitation can be efficiently overcome with tomography techniques that directly reconstruct the spatial distribution of tissue SoS in the field of view of US images. For this purpose, we have developed the computed ultrasound tomography in echo mode (CUTE) technique, which allows us to image tissue SoS using conventional hand-held pulse-echo US systems^29–32^. CUTE reconstructs the spatial distribution of tissue SoS by measuring local echo phase shifts caused by SoS heterogeneities when probing tissue at varying steering angles in transmission and reception. This is practically equivalent to having a virtual receiver that measures time delays between specific wave-propagation paths at every spatial location. So far, CUTE has proven excellent in retrieving correct SoS values with approximately 10 m/s contrast resolution in tissue-mimicking phantoms^33^, opening up the prospect of quantitative non-invasive diagnosis with SoS imaging.

This work presents a preliminary investigation of the performance of CUTE-based liver SoS estimates (CUTE-SoS) for diagnosing hepatic steatosis in an investigator-initiated, cross-sectional, first-in-human clinical study. Specifically, we evaluate the ability of CUTE-SoS to differentiate between normal livers and livers diagnosed with steatosis. The presence or absence of steatosis was diagnosed by combining conventional B-mode US and CAP, allowing us to recruit patients within the standard clinical routine. Our results show that liver CUTE-SoS estimates correlate strongly with CAP values and have excellent sensitivity and specificity for distinguishing normal and steatotic livers. This demonstrates that liver CUTE-SoS is a promising quantitative biomarker for diagnosing liver steatosis.

## Methods

### Study design and participants

This single-center, prospective, cross-sectional study was conducted at the Department for Visceral Surgery and Medicine at the Bern University Hospital (Inselspital Bern, Switzerland) between August 2021 and November 2021. This was a first-in-human phase II diagnostic study, meaning that its goal was to determine the diagnostic accuracy of liver CUTE-SoS estimates through a case-control study, including both participants with disease diagnosed by a standard method and healthy participants^34^. We recruited 22 patients with hepatic steatosis and 22 healthy individuals with no history of pre-existing liver conditions. Individuals with steatosis were recruited from patients who obtained their diagnosis during a regular screening visit at our center. This diagnosis is routinely performed using both conventional B-mode US images and CAP values. The former is based on the analysis of the parenchymal brightness, liver-to-kidney differences in echogenicity, posterior attenuation, and vessel blurring, which allow a semi-quantitative grading of steatosis (none, mild, moderate, or severe)^35^. Inclusion criteria for steatotic patients were: (i) indications of steatosis on B-mode US images, (ii) CAP values above 280 dB/m, a threshold showing over 90% sensitivity and positive predictive value for detecting steatosis^7^, and (iii) no fibrosis to avoid potential correlations with liver SoS^36^. Healthy volunteers were included if the absence of steatosis was confirmed in B-mode US images and had CAP values lower than 248 dB/m. All participants provided written informed consent in compliance with the principles of the Declaration of Helsinki and Good Clinical Practice. The study was approved by the Bern Cantonal Ethics Committee (ID 2020-03041). Study data were collected and managed using REDCap (Research Electronic Data Capture) hosted at the University of Bern^37,38^. REDCap is a secure, web-based software platform designed to support data capture for research studies.

CAP measurements used in the study were acquired in a second visit and were supported with B-mode US to confirm the assignment of participants to the two groups. During the same visit, we collected US radio-frequency signals required for CUTE-SoS analysis immediately after the B-mode US examination of each participant. A single physician experienced in US performed the data acquisition following a standardized acquisition protocol, with participants in the supine position, fasting state, and after at least five minutes of rest. Measurements corresponded to the right lobe liver parenchyma in the intercostal space. Although we integrated the CUTE-SoS imaging technology into the US system for clinical use, the physician was guided by conventional B-mode images and blinded to SoS images during the acquisition of US radio-frequency signals. These signals were stored in the US system and used for CUTE-SoS reconstructions by a researcher blinded to participants’ characteristics and CAP values.

### CAP measurements

We measured CAP values using the FibroScan 502 Touch (Echosens, Paris, France) transient elastography system with either the M or XL probe, which is automatically suggested by the device depending on the skin-to-capsule distance. The system provided the median (in dB/m) and inter-quantile range of a total of ten valid CAP measurements.

### Ultrasound system

The conventional US exploration and the data acquisition for CUTE-SoS imaging were performed using the SuperSonic® MACH® 30 US system (Hologic®–Supersonic Imagine®, Aix en Provence, France) with the C6-1X convex probe. It has a center frequency of 3.46 MHz, a curvature radius of 60.34 mm, and 192 transducer elements with 0.336 mm pitch size. CUTE-SoS imaging modality is not yet commercially available. Thus, we developed a fully integrated convex-probe CUTE implementation on the US system specifically for this study^32^. We used a Matlab-based framework (R2020b, MathWorks Inc., Natick, Massachusetts, USA) on a host computer to set up the parameters defining the scan sequence of the US transmission and signal reception. Loading of these parameters to the US system, triggering execution of the scan sequence, and raw data transfer back to the host computer was done via Ethernet connection. A dedicated graphical user interface was created on the host computer to simultaneously visualize the echo-intensity B-mode and CUTE-SoS images reconstructed from the transferred data using a Matlab implementation of the CUTE software. The data transfer and image reconstruction took approximately 20 seconds per acquisition. Since the physician collecting US radio-frequency signals was guided by conventional B-mode images and blinded to SoS images, we stored the data on the computer for an offline SoS reconstruction. Five acquisitions were taken per participant in order to use the mean liver CUTE-SoS values for the analysis.

### CUTE-SoS imaging

**Fig. 1:**
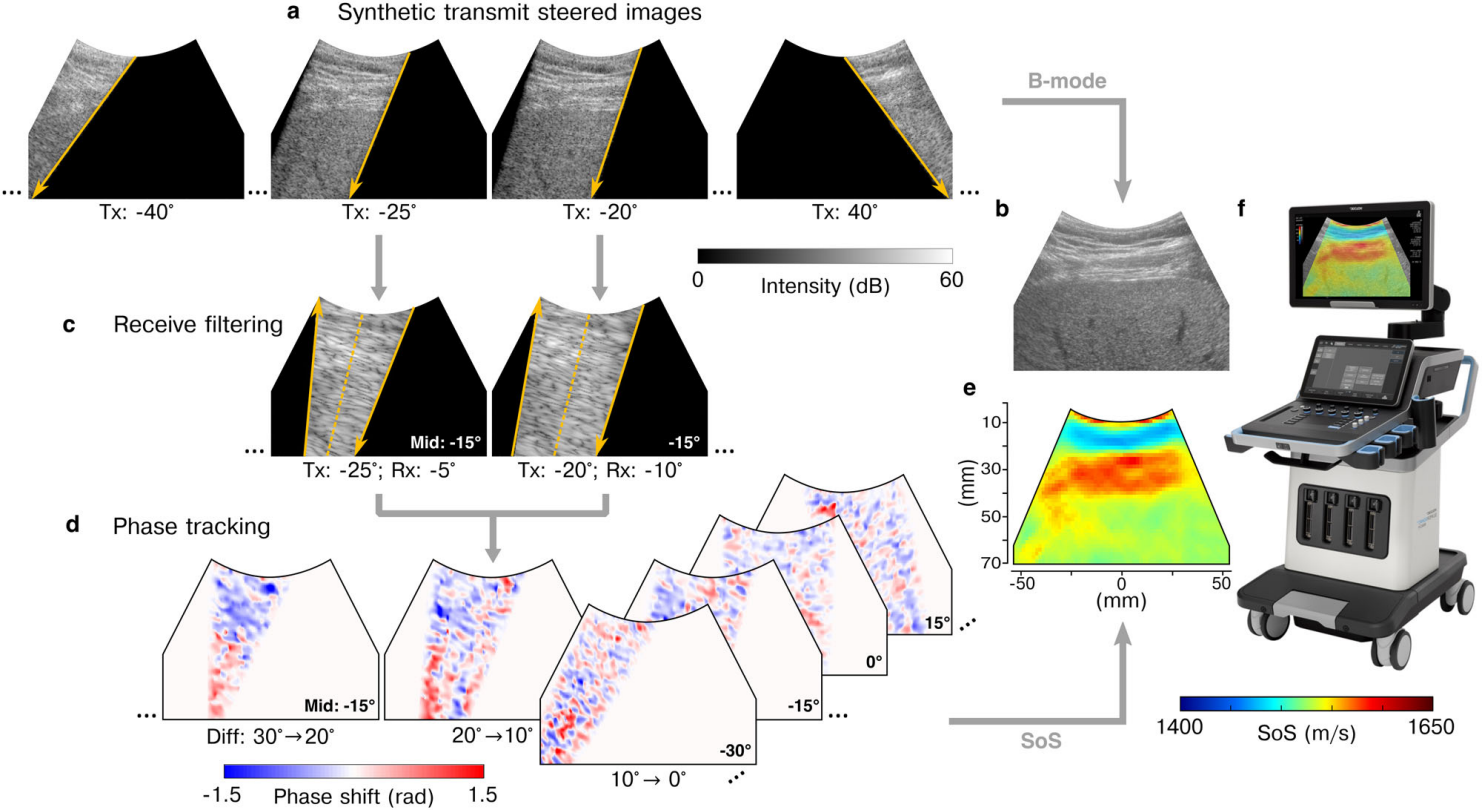
Computed ultrasound tomography in echo mode (CUTE) workflow. **a** Tissue is insonified by sequentially transmitting a set of wavefronts with varying steering angles. We use delay-and-sum beamforming and coherent compounding on the recorded radio-frequency signals to reconstruct synthetically focused and steered ultrasound images for transmit (Tx) angles ranging from −55° to 55° relative to the axial direction. Orange downwards-pointing arrows indicate the orientation of these Tx wavefronts. **b** We combine all these images to form a conventional echo-intensity B-mode image. **c** For CUTE, each image in (**a**) is decomposed via spatial frequency filtering into a set of images corresponding to different receive (Rx) steering angles (orange upwards-pointing arrows). These images are characterized by the mid angle (Mid) between Tx and Rx angles (dashed orange lines). Tx-Rx angle pairs with identical mid-angle provide well-correlated images. **d** We extract the spatial distribution of echo phase shifts from such well-correlated image pairs. We obtain a phase-shift map per mid angle and Tx-Rx angle difference (Diff). **e** Tissue speed of sound (SoS) is estimated from phase-shift maps solving a linear inverse problem. **f** The result is displayed together with the B-mode image in the SuperSonic® MACH® 30 ultrasound system (picture provided by SuperSonic Imagine® and adapted with permission). Although CUTE uses complex radio-frequency images in steps (**a**) and (**c**), here we show echo-intensity images for illustrative purposes.

Figure 1 illustrates the workflow of CUTE-SoS imaging. We acquired US signals using transmissions following a linear time delay law, similar to previous studies on CUTE^31,32^. With the convex probe, this linear delay law generates divergent waves with a constant transmit angle relative to the aperture surface normal^32^. We sequentially collected complex (analytic) radio-frequency signals at every transducer element for transmit angles ranging from −40° to 40° relative to the aperture surface normal, with a 1° angle step. For each transmit angle, we reconstructed the corresponding complex radio-frequency images using delay-and-sum beamforming with initial constant SoS of 1540 m/s on an image area of 75 mm in axial (*z*) by 100 mm in transversal (*x*) direction (Fig. 1a). Using coherent compounding, these images were then transformed into a new set of images where each one was synthetically steered on transmission along angles defined relative to the *z*-direction ranging from −55° to 55° with a 2.5° angle step. Note that this angle range can be larger than the range relative to the aperture surface normal due to the curvature of the probe. For each angle, the coherent compounding also provided synthetic focusing with an angular aperture of 5° to reduce clutter and grating lobes. Each resulting image was again transformed into a subset of images synthetically steered in reception using spatial frequency domain filtering^31^ for the same sequence of angles as in transmission (Fig. 1c). In this way, we obtained an image per pair of transmission and reception angle. We determined the spatial distribution of echo phase shifts using zero-lag complex cross-correlation between images of successive angle pairs having identical mid angle (Fig. 1d). Finally, we related the echo phase shift and tissue SoS at every spatial location assuming straight-ray propagation of US waves. The estimation of SoS from echo phase-shift measurements involves an ill-posed linear inverse problem; thus, we used first-order Tikhonov regularization defined along Cartesian coordinate axes to ensure meaningfully smooth solutions (Fig. 1e). This approach constrains the first-order spatial derivatives of variations in tissue slowness (i.e., inverse of SoS). While the derivatives could also be defined along polar coordinates to favor tissue structures following the curvature of the convex probe, the Cartesian Tikhonov regularization was found to provide higher accuracy in calibrated phantoms^32^. Following the notation in ref. ^32^, the regularization parameter values were fixed to *γ*_*x*_ = 13.1 and *γ*_*z*_ = 4.6, and the reconstruction grid size was 1.6 mm in the *x*-direction by 1.4 mm in the *z*-direction. The reader is referred to ref. ^32^ for further details about the technical aspects and implementation of the method.

### Statistical analysis

Statistical analysis and visualization were performed using Python (version 3.7.11) with the SciPy (1.6.2), Pandas (1.3.1), Seaborn (0.11.2), Pingouin (0.5.1), and Scikit-learn (1.0.1) libraries. Descriptive statistics including the mean, median, standard deviation, minimum and maximum values were used to summarize continuous variables, and frequencies and percentages were used for categorical variables. Here, we applied a two-tailed independent t-test to analyze the mean differences between two groups and Cohen’s *d* for calculating the effect size. The nonparametric two-sided Mann–Whitney *U* test was used to analyze the statistical significance of differences in distributions of CUTE-SoS and CAP in normal and steatotic livers, with the effect size computed via the standardized z-score. The correlation between CAP and CUTE-SoS measurements was calculated with the Pearson correlation coefficient. The degree of correlation and agreement between repeated CUTE-SoS measurements was assessed by the intraclass correlation coefficient using two-way random-effects model, average measure, and absolute agreement^39^. Results were considered significant for *p*-values lower than 0.05. The sensitivity and specificity of CUTE-SoS in predicting steatosis were analyzed using the area under the receiver operating characteristic (ROC) curve (AUC), with uncertainties computed by bootstrapping over 1000 realizations. The optimal SoS cut-off was determined by maximizing the geometric mean of the sensitivity and specificity.

### Reporting summary

Further information on research design is available in the Nature Portfolio Reporting Summary linked to this article.

## Results

### Study participants

In this study, we evaluated the liver of 44 participants, of which 22 were healthy volunteers, and 22 were patients diagnosed with steatosis. Their median (range) age and body mass index were 37 (24–77) years and 26 (19–38) kg/m^2^, respectively. Participants with liver steatosis were older (mean 47 years vs. 34 years, *p* = 0.002, Cohen’s *d* = 0.27) and more obese (30 kg/m^2^ vs. 23 kg/m^2^, *p* = 2.8 × 10^−10^, Cohen’s *d* = 0.65) than their healthy counterparts (Table 1).

**Table 1:** Characteristics of study population.

	Healthy	Steatotic
	*n* = 22	*n* = 22
Age (years)		
Mean (SD)	34 (10)	47 (15)
Min, max	24, 58	24, 77
Sex-*n*		
Female (%)	14 (63.6)	6 (27.3)
Male (%)	8 (34.4)	16 (72.7)
Weight (kg)		
Mean (SD)	70 (12)	94 (16)
Min, max	50, 99	65, 124
BMI (kg/m^*2*^*)*		
Mean (SD)	23 (2)	30 (4)
Min, max	19, 28	25, 38
CAP (dB/m)		
Mean (SD)	195 (26)	333 (28)
Min, max	160, 238	267, 394

*BMI* body mass index, *CAP* controlled attenuation paramater.

### Reference diagnosis and CAP measurements

Participants with liver steatosis were recruited from patients who obtained their diagnosis during a regular screening visit at the Bern University Hospital. For consistency, CAP measurements for this study were acquired in a second visit, right before the US data acquisition for CUTE-SoS. Although all patients had CAP ≥280 dB/m at the time of recruitment, one patient showed values below this threshold in the second visit (267 dB/m, Supplementary Table 1). Still, the patient was included in the study because feature analysis in conventional B-mode US images confirmed steatosis (see Methods section). The median of ten CAP measurements per participant ranged from 267 dB/m to 394 dB/m and from 160 dB/m to 238 dB/m for steatotic and normal livers, respectively (mean 333 dB/m vs. 195 dB/m, *p* = 7.16 × 10^−09^, *z* = 0.88, Table 1).

### CUTE-SoS images

US radio-frequency signals were acquired by a physician blinded to CUTE-SoS images to avoid biases. From a total of 220 acquisitions (five per participant), three resulted in empty datasets (1.4%), probably due to a systemic failure in the data storage process. Acquired radio-frequency signals were then used to reconstruct CUTE-SoS images and extract liver SoS values by a researcher blinded to participants’ characteristics and CAP values.

**Fig. 2:**
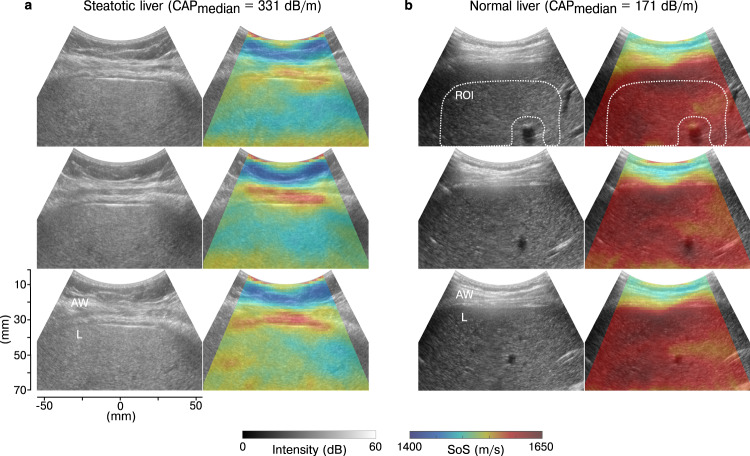
Example of ultrasound B-mode and CUTE speed of sound images in a steatotic and normal liver. **a**, **b** Echo intensity (left column) and speed-of-sound (SoS) distribution (right column) in a steatotic and normal liver with median controlled attenuation parameter (CAP) values of 331 dB/m and 171 dB/m, respectively. In each case, we show images corresponding to three distinct data acquisitions (rows) to illustrate the repeatability of CUTE-SoS and the correspondence between structures in B-mode and CUTE-SoS images. White dotted lines in the first row in (**b**) show an example of the manually selected region of interest (ROI) with blood vessels (dark areas) excluded, from which we extracted the mean liver SoS value. AW abdominal wall, L liver.

Figure 2 shows examples of B-mode and CUTE-SoS images in a normal and steatotic liver (all images in Supplementary Fig. 1). The two types of images display different tissue properties with complementary physiological and structural information. Reconstructed CUTE-SoS images show excellent axial resolution, capturing the SoS distribution of the abdominal wall and liver. The axial resolution is particularly high in the shallowest region, as suggested by the spatial correspondence between the reconstructed subcutaneous fat-intercostal muscle interface (low and high SoS, respectively) in B-mode and CUTE-SoS images (Fig. 2a). In contrast, the liver capsule generally appears more diffused, probably caused by tissue composition and architecture complexity of the abdominal wall region. This intercostal space contains muscles, connective tissues, fat inclusions, and highly reflective interfaces, which may introduce reverberation artifacts similar to those observed in elastography^40^, resulting in unreasonable SoS values within approximately the first 10 mm below the liver capsule. In the liver, fat accumulation reduces SoS values; thus, reconstructed SoS is generally lower in steatotic livers than in normal ones. In some cases, we observe large blood vessels that appear anechoic (dark) in B-mode images (Fig. 2b). Measured echo phase shifts within these regions are unreliable^41^ and may generate SoS artifacts inside and around the vessels (e.g., the lower SoS area on top of the blood vessels in Fig. 2b).

### Liver CUTE-SoS

**Fig. 3:**
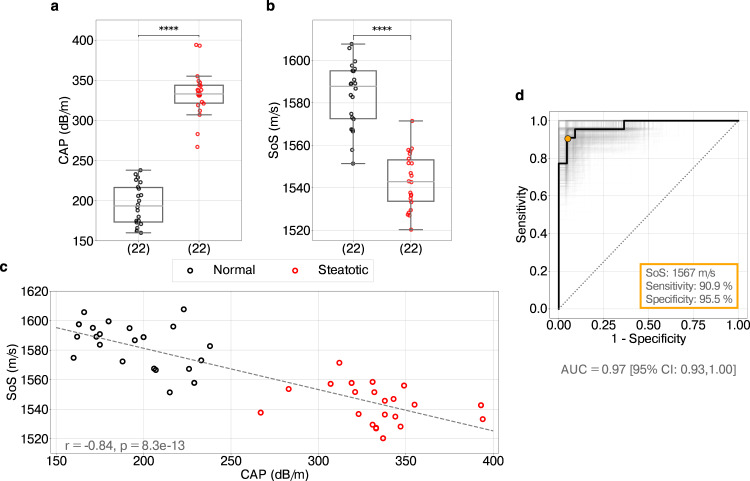
Discriminative performance of liver CUTE speed of sound. **a**, **b** Distribution of controlled attenuation parameter (CAP) and speed-of-sound (SoS) values in normal (black circles) and steatotic (red circles) livers, respectively (*n* = 22 biologically independent samples in each group). Box boundaries represent the first and third quartiles, the line across the box shows the median, and whiskers indicate the minimum and maximum values excluding outliers (values beyond 1.5 × inter-quartile range). The significance of differences between distributions is assessed with two-tailed Mann–Whitney *U* test, where **** refers to *p* < 0.0001. **c** Correlation between CAP and SoS values using Pearson’s r coefficient. The dashed line illustrates the predicted linear trend. **d** Receiver operating characteristic (ROC) curve representing true-positive rates (sensitivity) against false-positive rates (1−specificity) of predicting steatotic versus normal livers based on SoS values. The diagonal line indicates random prediction. The confidence interval (CI) is computed with bootstrapping, illustrated as different realizations of ROC curves in gray, with a total of 1000 realizations. The optimal SoS cut-off value is indicated in orange, with the corresponding sensitivity and specificity. AUC Area under the curve.

We extracted mean liver SoS values and corresponding standard deviations (Supplementary Table 1) by manually selecting regions of interest (ROI) that exclude the first 10 mm below the liver capsule and areas surrounding large blood vessels with approximately 5 mm extension to avoid reconstruction artifacts (Fig. 2b and Supplementary Fig. 1). The reliability of repeated mean SoS measurements (Supplementary Table 2) was excellent, with the intraclass correlation coefficient ranging between 0.95 and 0.98 (*p* = 4.08 × 10^−58^). Whereas patients with liver steatosis had significantly larger CAP values than healthy individuals (Fig. 3a), their mean liver SoS values were significantly lower (1543 m/s vs. 1584 m/s, *p* = 4.08 × 10^−08^, *z* = 0.89), ranging from 1520 m/s to 1571 m/s and from 1551 m/s to 1608 m/s for steatotic and normal livers, respectively (Fig. 3b). CAP and SoS values were therefore negatively and significantly correlated (*r* = − 0.84, *p* = 8.27 × 10^−13^) (Fig. 3c), meaning that both parameters are sensitive to variations in tissue composition. The mean variability of SoS within the ROI did not reveal any significant difference between the two groups (*p* = 0.21), ranging from 6 m/s to 13 m/s for steatotic livers and from 7 m/s to 13 m/s for normal ones (Supplementary Table 2). The average standard deviation was 10 m/s, in agreement with values observed in previous works^33^.

The receiver operating characteristic (ROC) curve analysis showed an excellent discriminative ability of liver CUTE-SoS to distinguish normal and steatotic livers (Fig. 3d), with an area under the curve (AUC) of 0.97 (95% confidence interval (CI): 0.93–1.0). The optimum SoS cute-off was 1567 m/s (95% CI: 1558–1573 m/s), which had a sensitivity of 90.9% (95% CI: 84–100%) and specificity of 95.5% (95% CI: 81–100%) for identifying patients with liver steatosis.

## Discussion

This first-in-human phase II diagnostic study demonstrates that liver CUTE-SoS is a promising quantitative biomarker for diagnosing liver steatosis non-invasively. CUTE is a recently developed US imaging modality capable of reconstructing the spatial distribution of tissue SoS using conventional handheld pulse-echo systems. It can thus be easily implemented in clinical US systems complementing other well-established modalities such as echo-intensity B-mode imaging, Doppler flow imaging, and US elastography. CUTE images allow efficient quantification of liver SoS by manually selecting an ROI within the organ guided by B-mode images to avoid reverberation and blood-vessel artifacts, offering a flexible diagnostic tool for physicians.

In this first clinical evaluation of CUTE technology, we designed a two-group study with healthy volunteers and steatotic patients where CAP in combination with B-mode US was used to assign participants to the two groups. We additionally used the inclusion criteria CAP≥280 dB/m (steatotic) and CAP < 248 dB/m (healthy) to ensure an accurate diagnosis of participants^7,20,42^. In this context, we found that liver CUTE-SoS shows high sensitivity (90.9%) and specificity (95.5%) for distinguishing the two groups with a cut-off SoS value of 1567 m/s. The seemingly reduced discriminating power of liver CUTE-SoS compared to CAP in Fig. 3a, b does not necessarily indicate an inferior diagnostic accuracy. Although CAP values have some uncertainty in quantifying liver fat content, here we used them as part of participant inclusion criteria; thus, an artificially perfect diagnostic performance of CAP is expected. Furthermore, we also found a very strong inverse correlation (*r* = −0.84) between liver CUTE-SoS and CAP values, consistent with the fact that US waves propagate slower and attenuate more in tissue with increased fat content. This result is valid independent of the diagnosis of individual subjects and may suggest that liver CUTE-SoS correlates to the gold standards quantifying steatosis similarly to CAP. However, the limited accuracy of CAP in determining the liver fat content^23^ does not allow us to draw conclusions on the ability of CUTE to grade steatosis.

The diagnostic potential of liver SoS for quantifying hepatic steatosis was recently observed in a pilot study by Dioguardi Burgio et al.^27^. For study cohorts considered in our work, they obtained an AUC ranging from 0.91 to 1.0, similar to our case (0.93–1.0). Their method, however, only provides an average liver SoS estimate and thus requires a manual correction of the subcutaneous layer thickness with assumed SoS values to avoid biases^25,28^. Furthermore, the US acquisition needs to carefully avoid the presence of large hepatic vessels and place the probe parallel to the liver capsule^27^. In comparison, CUTE is more efficient and provides a complete picture of the tissue composition through spatially resolved SoS images. This spatial resolution is crucial to extend the clinical use of CUTE beyond the assessment of hepatic steatosis and honor the versatility of US imaging modalities. For instance, in liver applications, the spatial resolution of CUTE-SoS images may in the future help identify focal liver lesions such as liver adenomas and hepatocellular carcinoma. In principle, CUTE can be employed in any organ accessible to pulse-echo US; thus, its clinical applications may also extend to breast cancer diagnosis^43–45^ or the assessment of musculoskeletal disorders^26,46,47^, where SoS has already proven to be clinically relevant. Furthermore, CUTE-SoS images can also be used to correct aberrations in conventional B-mode images and improve their quality^48^.

The diagnostic accuracy of liver CUTE-SoS ultimately depends on the relationship between the liver fat content and the actual liver SoS. Hence, understanding how the latter relates to liver CUTE-SoS estimates is critical. Although the accuracy of absolute CUTE-SoS values is difficult to evaluate without knowing the ground truth values, liver CUTE-SoS estimates are in excellent agreement with SoS measurements in excised human livers reported in the literature. For normal livers, reported values range from 1560 m/s to 1607 m/s^49–51^, whereas CUTE-SoS values in this study range from 1551 m/s to 1608 m/s. These values decrease in steatotic livers, where the minimum reported SoS value is 1522 m/s^49^ compared to the 1520 m/s that we estimate. This consistency suggests that CUTE can estimate the correct liver SoS value, which is key to quantifying the fat content in the liver non-invasively^27,52^. However, in vivo liver SoS values could, in principle, deviate from those in excised livers, and comparing our results to other reported in vivo values may be more appropriate. For healthy volunteers, Imbault et al.^25^ estimated SoS values ranging from approximately 1530 m/s to 1605 m/s, while Boozari et al.^36^ found values between 1540 m/s and 1580 m/s, and Dioguardi Burgio et al.^27^ reported values from 1525 m/s to 1625 m/s. Our liver CUTE-SoS estimates are within these values, although they show a narrower range. For steatotic patients, the minimum liver SoS value found in these studies is 1460 m/s^27^, considerably lower than in our case. While partial consistency is necessary, we do not expect an exact agreement between in vivo liver SoS values estimated using different techniques. The reason is that different results can be prone to different biases that depend on the specific nature of each technique.

As discussed in previous studies, several factors can influence the quality of CUTE-SoS reconstructions. In the convex-probe CUTE implementation, Jaeger et al.^32^ observed that liver CUTE-SoS estimates are increasingly biased with depth towards the initial SoS used for beamforming, probably due to US wavefield aberrations caused by the abdominal wall and the increasingly reduced data coverage with depth. To minimize this bias, in this study, we adopted the following strategies: (i) Unlike in ref. ^32^, where CUTE images are obtained near the linea alba, here we scanned the liver through the intercostal space, which is the clinical standard for liver US because it minimizes aberrations. (ii) We limited the maximum imaging depth to 70 mm as opposed to 100 mm used in ref. ^32^. (iii) We fixed the initial beamforming SoS value to 1540 m/s, standard in US imaging, to avoid biases when comparing relative liver CUTE-SoS variations between participants. While the choice of the beamforming SoS value is somehow arbitrary, this value did not particularly influence the discriminative ability of liver CUTE-SoS to distinguish normal and steatotic livers (Supplementary Fig. 2a). This means that potential biases introduced by the beamforming SoS do not affect the accuracy of relative liver CUTE-SoS variations detected between participants. The optimal cut-off value, however, can change and should be carefully interpreted. For instance, we observed variations of 15 m/s in the cut-off liver CUTE-SoS value for beamforming SoS values ranging from 1510 m/s to 1570 m/s (Supplementary Fig. 2a), approximately within the 95% CI of the cut-off value reported in this study. As is common in ill-conditioned inverse problems, CUTE-SoS reconstructions are also affected by the choice of the regularization approach and its strength^32,33^. Regularization is inherently subjective, and different approaches could lead to different liver CUTE-SoS values. For instance, Jaeger et al.^32^ compared first-order Tikhonov regularization defined along Cartesian or polar coordinates and showed that the former provides quantitatively more accurate results in phantoms. Following these results, and in the absence of in vivo ground truth SoS values, we chose to implement Cartesian regularization in this study. In the future, however, it would be interesting to compare different techniques, e.g., the L1-norm regularization^46^ or the Bayesian regularization^33^, to understand the precision in distinguishing healthy and steatotic livers. For first-order Tikhonov regularization, large regularization parameter values can lead to excessively smoothed reconstructions, blurring CUTE-SoS estimates at tissue interfaces, whereas small values can lead to oscillatory artifacts caused, for instance, by phase-shift noise. When varying the regularization parameters around the values used in this study, we did not find important changes in our results, especially in the discriminative ability of liver CUTE-SoS (Supplementary Fig. 2b). The main conclusions of this study are therefore insensitive to these possible sources of biases, strengthening the potential of liver CUTE-SoS for steatosis assessment.

The CUTE technique rests on the assumption that detected echo phase shifts are linearly related to tissue SoS variations. Under this assumption, liver CUTE-SoS estimates are independent of the diameter of the fat layer. In reality, however, some degree of nonlinearity is expected, although our results suggest that this was sufficiently weak to provide meaningful results with CUTE in this study. For instance, in cases where healthy volunteers and patients had a similar abdominal wall thickness, we obtained substantially different liver CUTE-SoS values, consistent with the reference diagnosis (Supplementary Fig. 1). Nevertheless, CUTE may experience technical difficulties in highly attenuating media due to the decrease in signal-to-noise ratio, similar to other US-based techniques. This can occur in patients with elevated BMI and thus a large subcutaneous fat layer thickness. Such patients would also require an increased imaging depth, which may reduce the robustness of liver CUTE-SoS estimates, as observed in ref. ^32^. In this study, the maximum BMI of 38 kg/m^2^ still did not pose major technical difficulties for estimating liver CUTE-SoS.

We provide preliminary evidence on the clinical value of CUTE in a single-center, two-group study with a relatively small and homogeneous study population. Our results, while promising, may depend on the selection criteria used for defining the two study groups; thus, they must be carefully interpreted. Healthy and steatotic groups were defined by combining B-mode US and CAP. This is not the gold standard for grading steatosis, although it shows high accuracy in detecting steatosis (our study purpose) for the CAP thresholds used here^7,19,20,42,53–55^. To assess the ability of CUTE to grade steatosis and monitor changes over time, a comparison with the reference standards MR proton density fat fraction (MRI-PDFF) or histology is required^23^. While being beyond the scope of this study, a larger and more diverse population would allow us to analyze the effects of confounding variables on liver CUTE-SoS estimates. For instance, these may include the BMI, abdominal wall thickness, or other disease types, such as liver fibrosis, which has been reported to increase liver SoS^36^. Repeated CUTE-SoS measurements showed excellent correlation and agreement between them. However, careful repeatability and reproducibility analysis are still needed to understand measurement variability comprehensively. Due to the lack of clinical experience with CUTE and to avoid biases in the data acquisition, we did not implement any quality criteria for data selection. A study with a larger cohort size will also allow us to investigate automatic criteria for prospectively including or excluding data sets, as is regularly done with other techniques. Such criteria can be based on the quality of B-mode images (e.g., presence of tissue motion or reverberation artifacts), echo phase-shift maps (e.g., phase-shift noise level), or CUTE-SoS images (e.g., uniformity of liver CUTE-SoS values).

In conclusion, CUTE offers a new quantitative imaging modality adapted to clinical US systems with promising diagnostic value for screening for NAFLD cost-efficiently and non-invasively. It can be routinely performed together with the conventional US exploration with no additional cost for the practitioner, helping to tackle the high clinical burden that the increasing prevalence of NAFLD involves.

### Supplementary information

Supplementary Information

Supplementary Data 1

Reporting summary

## Data Availability

Data supporting the statistical analysis in this article is accessible within the manuscript and Supplementary Information. Supplementary Data 1 contains source data underlying Fig. 3. These can also be downloaded via BORIS, the institutional repository of the University of Bern, at ref. ^56^. Anonymized ultrasound raw datasets collected and analyzed during this study are available from the corresponding author upon reasonable request. However, data containing identifiable patient information is considered confidential, and disclosure to third parties is prohibited.

## Code availability

The Python-based custom code for statistical analysis and generating Fig. 3 is publicly available at ref. ^56^. The custom code for CUTE-SoS image reconstruction is available from the corresponding author upon request, subject to specific agreements.
