# Supplementary material for: First-in-human diagnostic study of hepatic steatosis with computed ultrasound tomography in echo mode
Source: Commun Med (Lond). 2023 Dec 9;3:176. doi: 10.1038/s43856-023-00409-3 (PMC10710459; doi:10.1038/s43856-023-00409-3)
Supplement: Supplementary file 1 — Supplementary Information [file 43856_2023_409_MOESM1_ESM.pdf]

# First-in-human diagnostic study of hepatic steatosis with computed ultrasound tomography in echo mode

Patrick Stähli[1,4], Chiara Becchetti[2,4], Naiara Korta Martiartu[1,4], Annalisa Berzigotti[2, 3], Martin Frenz[1], and Michael Jaeger[1,*]

[1]Institute of Applied Physics, University of Bern, Bern, Switzerland.
[2]Department of Visceral Surgery and Medicine, Inselspital, Bern University Hospital, University of Bern, Bern, Switzerland.
[3]Department of Biomedical Research, University of Bern, Bern, Switzerland.
[4]These authors contributed equally.
[*]email: michael.jaeger@unibe.ch

## Content

This supplementary material provides measured CAP values, reconstructed CUTE-SoS images, and extracted liver CUTE-SoS values for each participant. Data acquisition for CUTE-SoS reconstruction was repeated five times; thus, we show all reconstructed CUTE-SoS images and their corresponding liver SoS values. Furthermore, we analyze the sensitivity of the discriminative performance of liver CUTE-SoS estimates to the initial beamforming SoS value and the strength of regularization parameters used in the inversion.

# Supplementary Tables

Supplementary Table 1: Data used for statistical analysis in this study. CAP: controlled attenuation parameter; IQR: interquartile range; CUTE: computed ultrasound tomography in echo mode; SoS: speed of sound; SD: standard deviation.

| Participant ID | Diagnosis | CAP (IQR) [dB/m] | CUTE-SoS (SD) [m/s] |
|---|---|---|---|
| CL-005 | Normal | 207 (27) | 1567 (12) |
| CL-007 | Normal | 180 (27) | 1600 (8) |
| CL-008 | Normal | 175 (15) | 1584 (5) |
| CL-009 | Normal | 215 (22) | 1551 (11) |
| CL-010 | Normal | 162 (21) | 1589 (16) |
| CL-011 | Normal | 188 (33) | 1572 (14) |
| CL-012 | Normal | 206 (30) | 1568 (3) |
| CL-013 | Normal | 229 (30) | 1558 (6) |
| CL-014 | Normal | 163 (81) | 1598 (10) |
| CL-015 | Normal | 166 (46) | 1606 (9) |
| CL-016 | Normal | 226 (18) | 1567 (14) |
| CL-017 | Normal | 238 (36) | 1583 (7) |
| CL-018 | Steatotic | 333 (37) | 1527 (11) |
| CL-019 | Normal | 171 (22) | 1595 (11) |
| CL-020 | Steatotic | 349 (28) | 1556 (9) |
| CL-021 | Normal | 200 (48) | 1589 (12) |
| CL-022 | Normal | 173 (25) | 1589 (6) |
| CL-026 | Normal | 160 (32) | 1575 (11) |
| CL-027 | Normal | 233 (10) | 1573 (5) |
| CL-028 | Normal | 217 (12) | 1596 (20) |
| CL-029 | Steatotic | 337 (31) | 1520 (20) |
| CL-030 | Steatotic | 332 (33) | 1552 (5) |
| CL-031 | Steatotic | 319 (33) | 1558 (5) |
| CL-032 | Steatotic | 307 (15) | 1557 (13) |
| CL-033 | Normal | 195 (28) | 1587 (4) |
| CL-034 | Normal | 175 (60) | 1591 (6) |
| CL-035 | Steatotic | 312 (40) | 1572 (11) |
| CL-036 | Steatotic | 355 (42) | 1543 (7) |
| CL-038 | Steatotic | 338 (44) | 1546 (3) |
| CL-039 | Steatotic | 394 (14) | 1533 (5) |
| CL-040 | Steatotic | 283 (11) | 1554 (2) |
| CL-041 | Steatotic | 331 (15) | 1530 (6) |
| CL-042 | Steatotic | 347 (30) | 1528 (7) |
| CL-043 | Normal | 223 (33) | 1608 (9) |
| CL-045 | Steatotic | 344 (13) | 1535 (9) |
| CL-046 | Steatotic | 331 (15) | 1558 (10) |
| CL-047 | Steatotic | 393 (39) | 1543 (5) |
| CL-050 | Steatotic | 321 (42) | 1552 (8) |
| CL-051 | Steatotic | 333 (38) | 1527 (7) |
| CL-052 | Steatotic | 323 (79) | 1537 (11) |
| CL-053 | Steatotic | 267 (14) | 1538 (6) |
| CL-054 | Steatotic | 343 (23) | 1547 (2) |
| CL-055 | Steatotic | 338 (35) | 1536 (11) |
| CL-056 | Normal | 192 (36) | 1595 (9) |

Supplementary Table 2: Mean and standard deviation of liver speed of sound (in m/s) per participant and ultrasound data acquisition (Acq.).

| **Participant ID** | **Acq. 1** | **Acq. 2** | **Acq. 3** | **Acq. 4** | **Acq. 5** |
|---|---|---|---|---|---|
| CL-005 | 1574±7 | 1572±8 | 1564±7 | 1546±10 | 1577±7 |
| CL-007 | 1587±14 | 1605±9 | 1599±10 | 1600±9 | 1607±9 |
| CL-008 | 1583±14 | 1578±8 | 1582±10 | 1580±8 | 1591±10 |
| CL-009 | 1539±7 | 1543±13 | 1557±18 | 1565±13 | 1553±14 |
| CL-010 | 1586±12 | 1570±14 | 1609±11 | 1591±8 | – |
| CL-011 | 1554±12 | 1592±7 | 1577±8 | 1575±9 | 1564±9 |
| CL-012 | 1566±10 | 1572±9 | 1569±7 | 1568±6 | 1563±9 |
| CL-013 | 1560±7 | 1557±14 | 1560±14 | 1547±11 | 1565±10 |
| CL-014 | 1589±11 | 1611±9 | 1592±8 | 1590±13 | 1605±10 |
| CL-015 | 1606±11 | 1603±7 | 1597±7 | 1604±8 | 1609±9 |
| CL-016 | 1555±10 | 1562±12 | 1556±10 | 1581±5 | 1583±10 |
| CL-017 | 1571±5 | 1581±13 | 1586±14 | 1591±16 | 1588±14 |
| CL-018 | 1528±15 | 1530±11 | 1542±8 | 1514±16 | 1521±11 |
| CL-019 | 1600±10 | 1598±14 | 1602±12 | 1600±14 | 1576±17 |
| CL-020 | 1544±8 | 1560±7 | 1548±9 | 1561±6 | 1566±9 |
| CL-021 | 1573±8 | 1582±10 | 1590±12 | 1602±11 | 1597±13 |
| CL-022 | 1595±7 | 1591±11 | 1590±9 | 1579±11 | 1590±7 |
| CL-026 | 1577±17 | 1587±9 | 1583±9 | 1564±12 | 1563±14 |
| CL-027 | 1581±7 | 1571±5 | 1572±7 | 1572±6 | 1567±10 |
| CL-028 | 1561±14 | 1599±10 | 1607±7 | 1610±8 | 1604±8 |
| CL-029 | 1497±10 | 1526±16 | 1524±15 | 1549±7 | 1506±10 |
| CL-030 | 1557±8 | 1552±5 | 1545±6 | 1548±6 | 1555±5 |
| CL-031 | 1562±7 | 1550±6 | 1563±9 | 1555±8 | 1558±7 |
| CL-032 | 1535±7 | 1570±9 | 1561±10 | 1558±7 | 1563±5 |
| CL-033 | 1592±9 | 1588±7 | 1584±18 | 1582±11 | 1588±12 |
| CL-034 | 1580±14 | 1594±8 | 1592±8 | 1597±7 | 1592±6 |
| CL-035 | 1556±8 | 1586±9 | 1571±6 | 1576±10 | 1568±11 |
| CL-036 | 1542±13 | 1556±12 | 1539±9 | 1540±10 | 1538±9 |
| CL-038 | 1546±8 | 1544±12 | 1549±12 | 1543±10 | 1547±9 |
| CL-039 | 1537±12 | 1528±13 | 1532±11 | 1530±12 | 1539±9 |
| CL-040 | 1554±8 | 1551±7 | 1552±6 | 1555±8 | 1556±11 |
| CL-041 | 1525±16 | 1526±13 | 1538±14 | 1530±14 | – |
| CL-042 | 1529±15 | 1524±16 | 1519±8 | 1539±7 | 1529±12 |
| CL-043 | 1608±13 | 1604±11 | 1599±22 | 1620±13 | – |
| CL-045 | 1548±7 | 1526±13 | 1539±7 | 1528±12 | 1535±10 |
| CL-046 | 1545±6 | 1555±8 | 1570±9 | 1554±9 | 1568±7 |
| CL-047 | 1538±7 | 1547±8 | 1549±8 | 1541±7 | 1537±7 |
| CL-050 | 1552±6 | 1550±10 | 1540±10 | 1563±8 | 1553±15 |
| CL-051 | 1532±6 | 1517±6 | 1525±4 | 1533±5 | 1530±9 |
| CL-052 | 1548±10 | 1548±11 | 1528±8 | 1536±9 | 1524±6 |
| CL-053 | 1542±13 | 1529±8 | 1536±5 | 1546±9 | 1535±8 |
| CL-054 | 1547±13 | 1548±10 | 1544±15 | 1546±13 | 1549±13 |
| CL-055 | 1537±21 | 1530±10 | 1521±14 | 1547±7 | 1547±10 |
| CL-056 | 1590±14 | 1583±16 | 1603±9 | 1602±12 | 1604±8 |

# Supplementary Figures

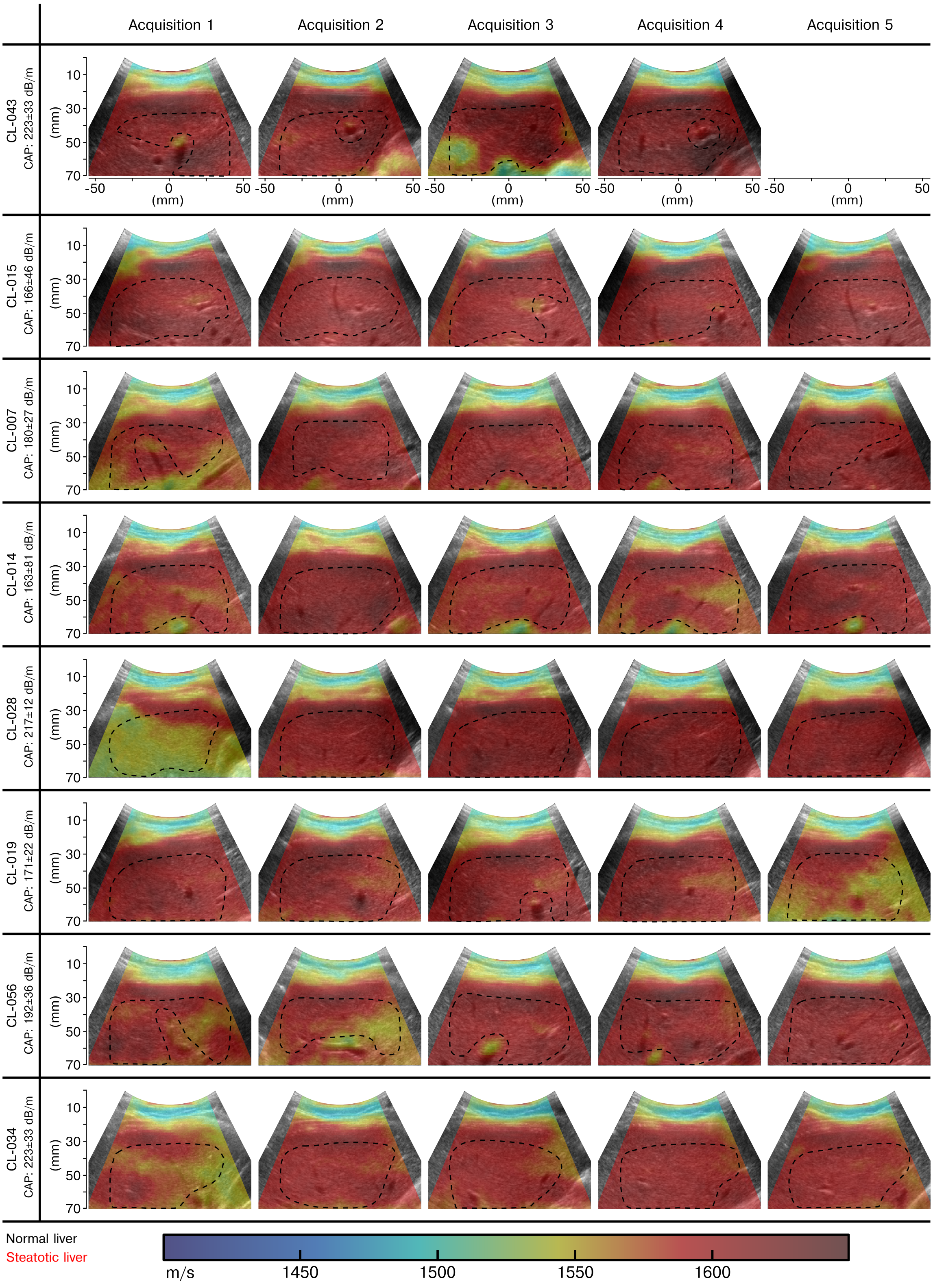


Supplementary Figure 1: Reconstructed speed-of-sound (SoS) images for each participant overlaid on B-mode images. Rows and columns indicate different participants and acquisitions, respectively. Participants appear in descending order in terms of liver SoS values, with the median and interquartile range of controlled attenuation parameter (CAP) values indicated in each case, as well as the diagnostic status. Dashed lines show manually selected regions of interest where mean liver SoS values are calculated. Empty spaces correspond to invalid acquisitions.

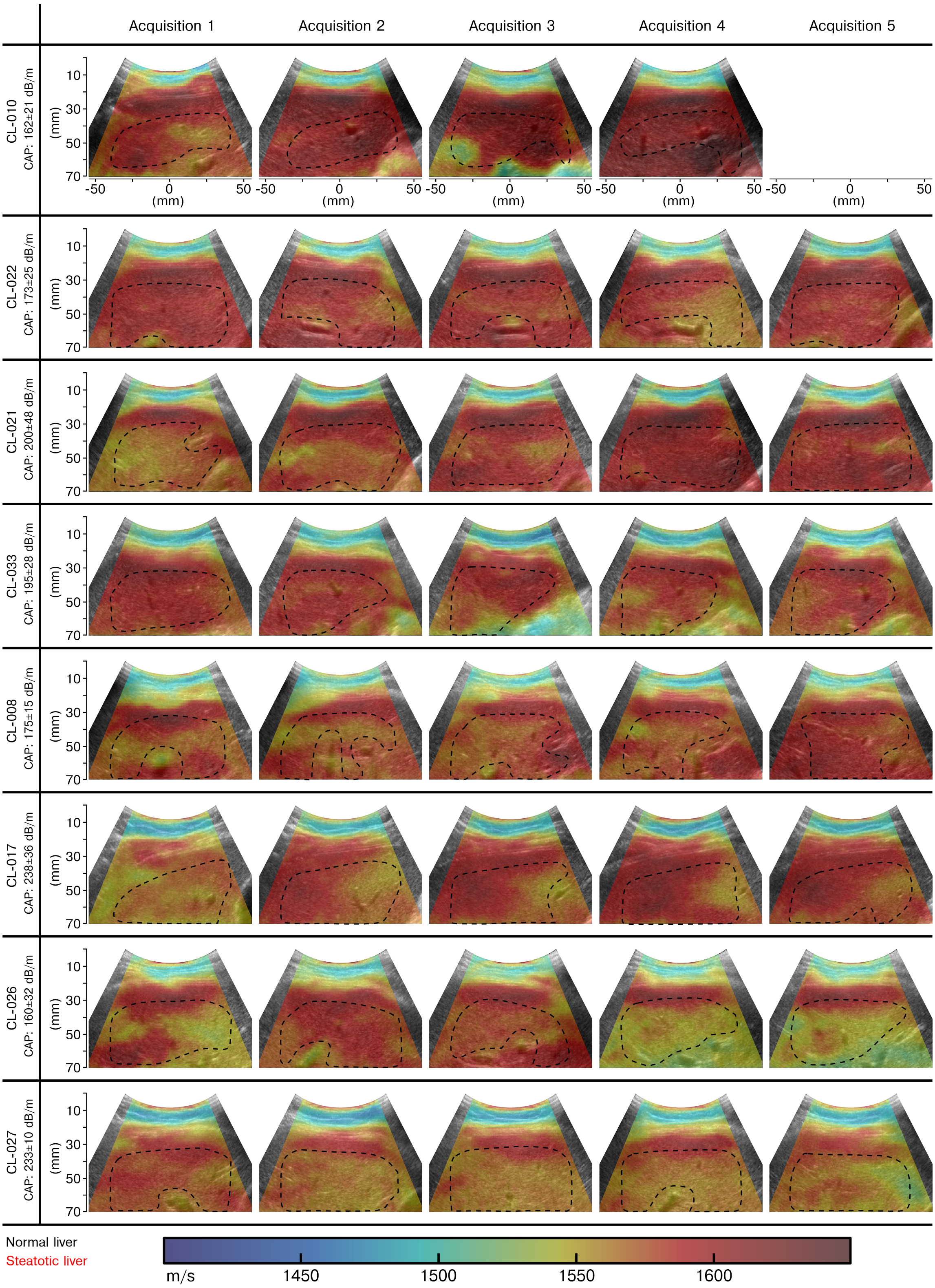


Supplementary Figure 1: (Continued) Reconstructed speed-of-sound (SoS) images for each participant overlaid on B-mode images. Rows and columns indicate different participants and acquisitions, respectively. Participants appear in descending order in terms of liver SoS values, with the median and interquartile range of controlled attenuation parameter (CAP) values indicated in each case, as well as the diagnostic status. Dashed lines show manually selected regions of interest where mean liver SoS values are calculated. Empty spaces correspond to invalid acquisitions.

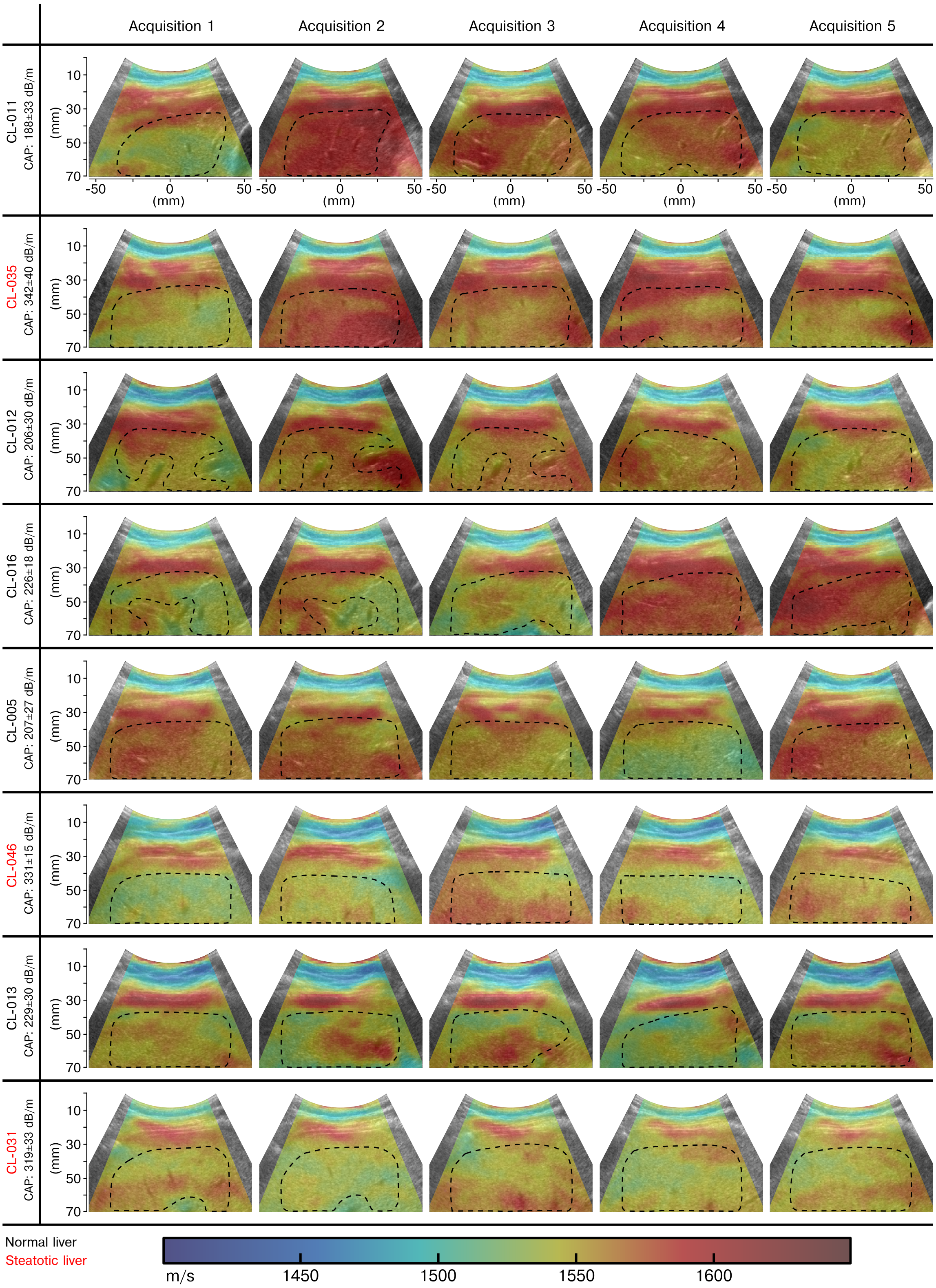


Supplementary Figure 1: (Continued) Reconstructed speed-of-sound (SoS) images for each participant overlaid on B-mode images. Rows and columns indicate different participants and acquisitions, respectively. Participants appear in descending order in terms of liver SoS values, with the median and interquartile range of controlled attenuation parameter (CAP) values indicated in each case, as well as the diagnostic status. Dashed lines show manually selected regions of interest where mean liver SoS values are calculated. Empty spaces correspond to invalid acquisitions.

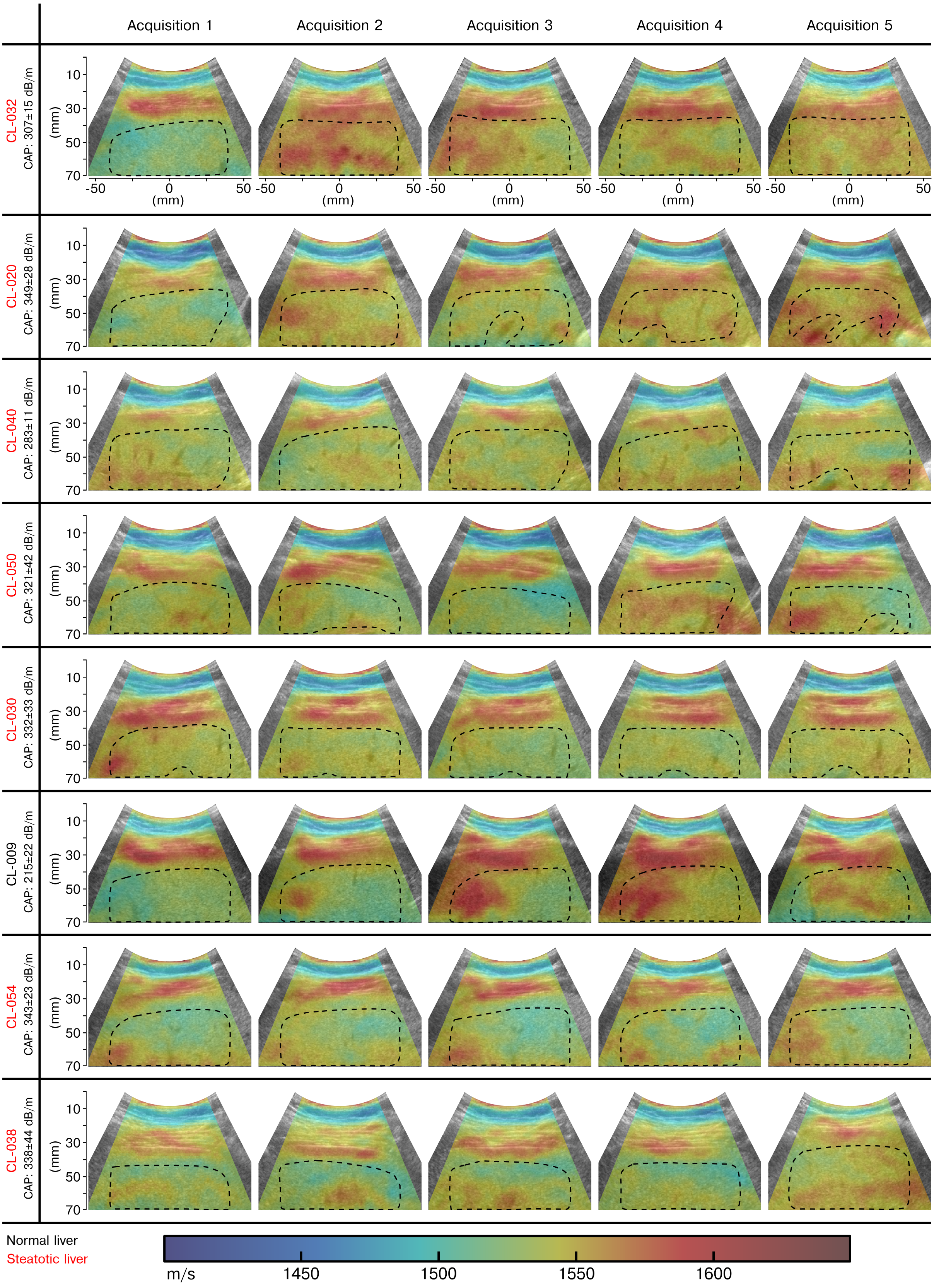


Supplementary Figure 1: (Continued) Reconstructed speed-of-sound (SoS) images for each participant overlaid on B-mode images. Rows and columns indicate different participants and acquisitions, respectively. Participants appear in descending order in terms of liver SoS values, with the median and interquartile range of controlled attenuation parameter (CAP) values indicated in each case, as well as the diagnostic status. Dashed lines show manually selected regions of interest where mean liver SoS values are calculated. Empty spaces correspond to invalid acquisitions.

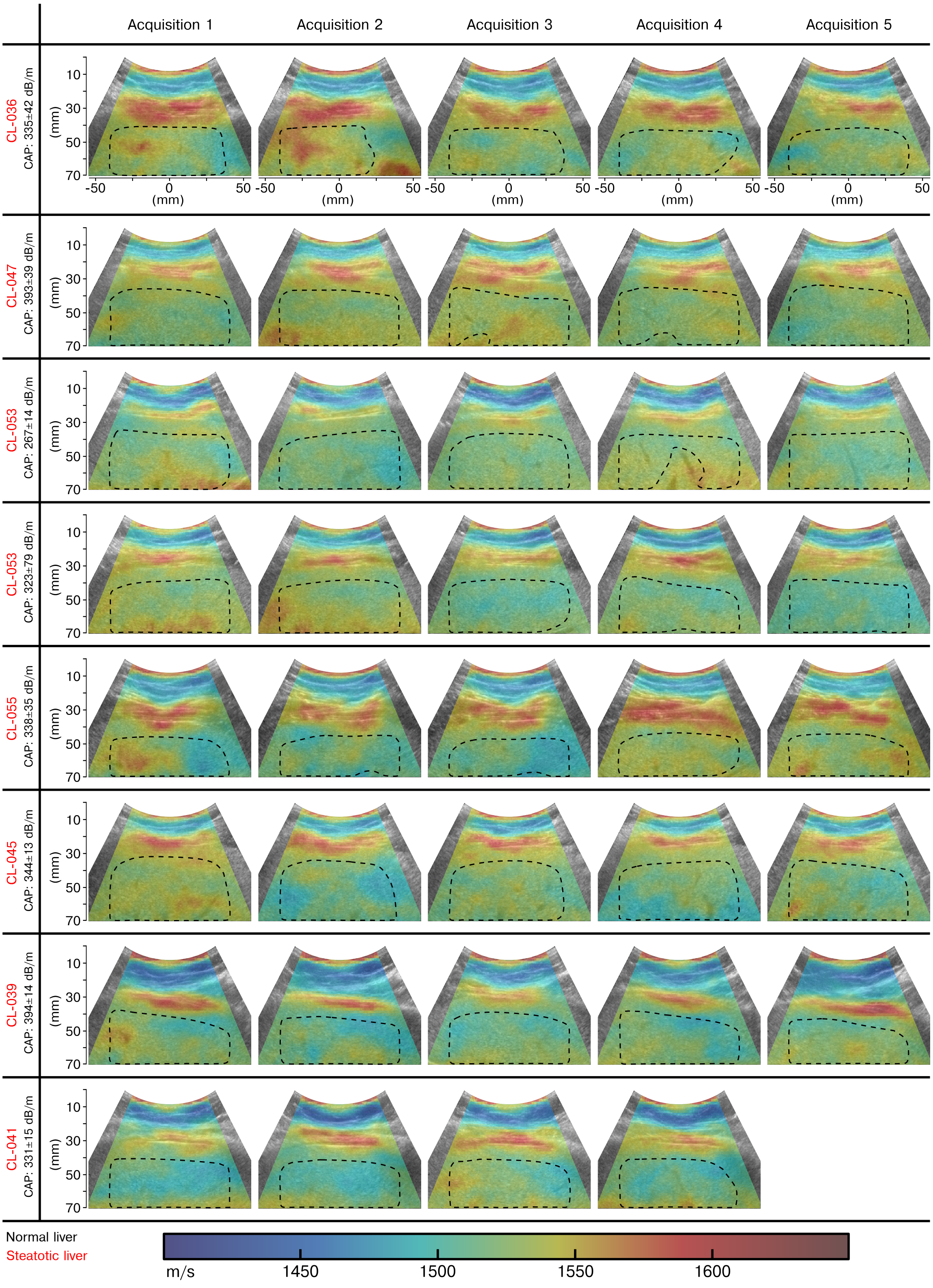


Supplementary Figure 1: (Continued) Reconstructed speed-of-sound (SoS) images for each participant overlaid on B-mode images. Rows and columns indicate different participants and acquisitions, respectively. Participants appear in descending order in terms of liver SoS values, with the median and interquartile range of controlled attenuation parameter (CAP) values indicated in each case, as well as the diagnostic status. Dashed lines show manually selected regions of interest where mean liver SoS values are calculated. Empty spaces correspond to invalid acquisitions.

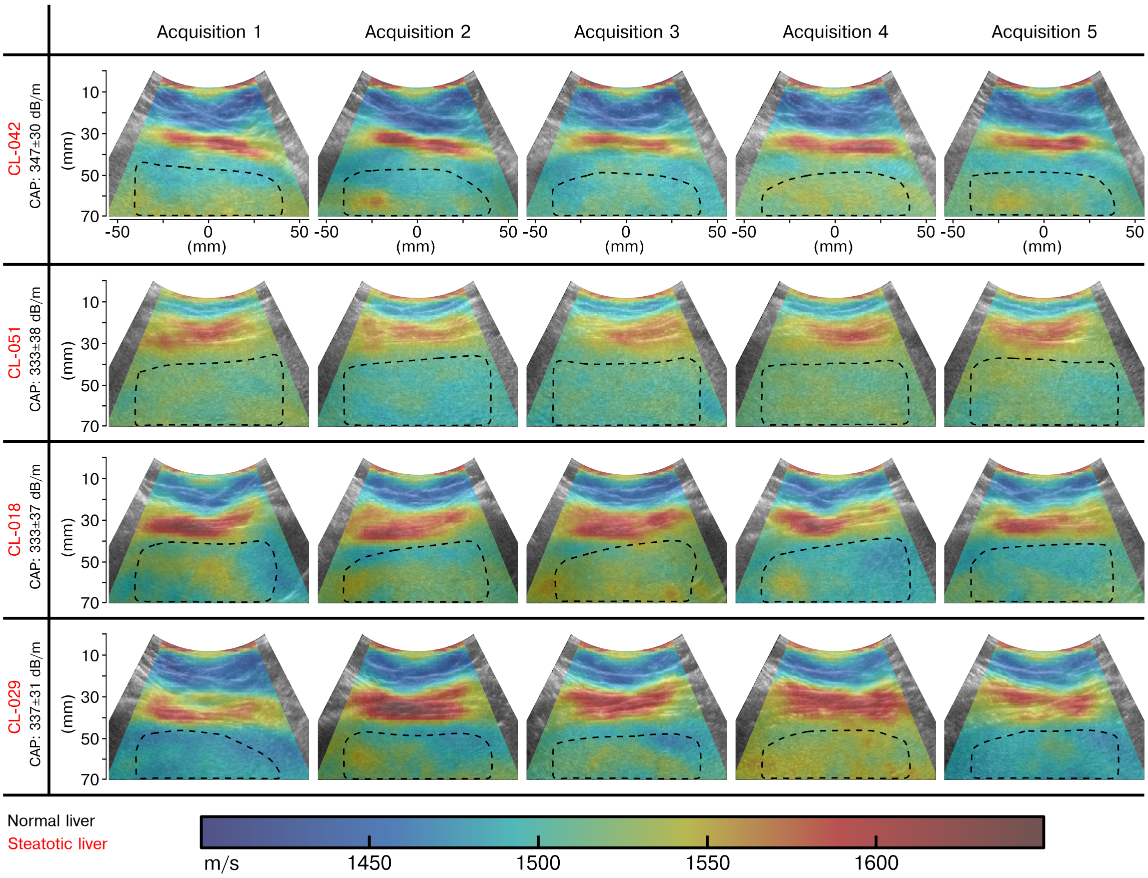


Supplementary Figure 1: (Continued) Reconstructed speed-of-sound (SoS) images for each participant overlaid on B-mode images. Rows and columns indicate different participants and acquisitions, respectively. Participants appear in descending order in terms of liver SoS values, with the median and interquartile range of controlled attenuation parameter (CAP) values indicated in each case, as well as the diagnostic status. Dashed lines show manually selected regions of interest where mean liver SoS values are calculated. Empty spaces correspond to invalid acquisitions.

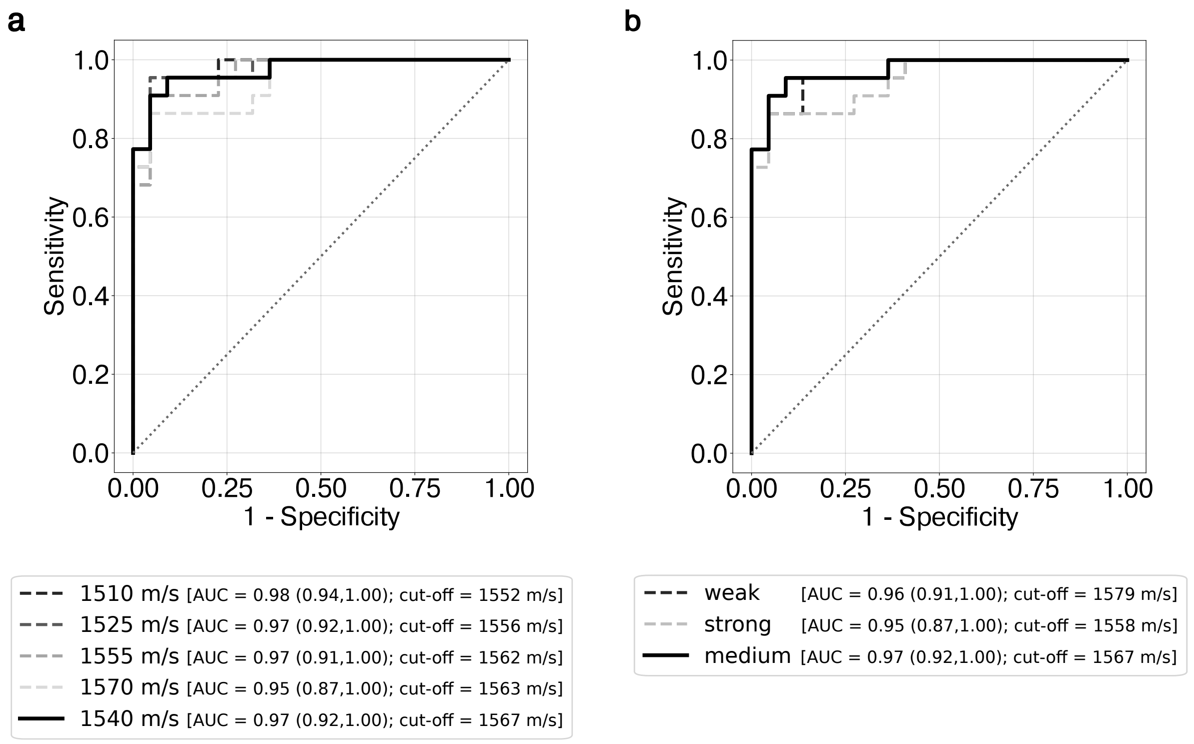


Supplementary Figure 2: Sensitivity of the discriminative performance of liver CUTE-SoS to initial SoS and regularization parameters. **a** Receiver operating characteristic (ROC) curve analysis for liver CUTE-SoS estimates that use initial beamforming SoS values ranging from 1510 m/s to 1570 m/s. The initial SoS value used in the main manuscript is indicated in black. **b** ROC curve analysis for different strengths of regularization parameter values. The results in the main manuscript were obtained with the regularization strength indicated in black (medium). The weak regularization divides by 1.3 the regularization parameter values used in the main manuscript, while the strong regularization multiplies them by 1.4. These factors were chosen to represent the full range of smoothing strengths while maintaining an acceptable trade-off between the spatial resolution and artifact level in CUTE-SoS images. AUC: Area under the curve; cut-off: optimal cut-off liver CUTE-SoS value.
